# Exploring student teachers’ perceptions of assessment ethics across university-based teacher education programs in Iran

**DOI:** 10.1186/s40468-022-00205-1

**Published:** 2023-02-13

**Authors:** Ali Darabi Bazvand

**Affiliations:** Department of English, Catholic University in Erbil, Erbil, Iraq

**Keywords:** Assessment ethics, Educational assessment, Ethical issues, Student teacher, Teacher education

## Abstract

It is acknowledged that assessment ethics is an integral part of teacher education. Unlike sizable published research on students' perceptions of assessment in higher education, very little is reported on student teachers' perceptions of assessment ethics across university-based teacher education programs. This study aimed to explore Teaching English as a Foreign Language (TEFL) student teachers’ perceptions of assessment ethics in the classroom. Qualitative (phone interview) data from 15 TEFL teacher candidates were collected and analyzed using thematic content analysis. Based on the informants' responses, three overarching issues in assessment ethics emerged and are namely related to (a) assessment development (i.e., content underrepresentation, one-dimensional assessment, surprise items), (b) assessment administration(i.e., time, noise, and inconsistency in educators' behaviors), and (c) assessment scoring and communication (i.e., lack of transparency in feedback provision' 'misalignment of grading practice,' and 'breaching confidentiality in grade communication'). Furthermore, it was revealed that assessment is, for a great part, teacher-initiated and summative-oriented. In light of these findings, this study can inform professional development programs on assessment in teacher education.

## Introduction

It is acknowledged that assessment ethics is an integral part of teacher education (Bergman, 2018; Maxwell & Schwimmer, 2016). Unlike sizable published research on students’ perceptions of assessment in higher education, until recently, little has been written on student teachers’ perceptions of assessment ethics across university-based teacher education programs (Bergman, 2013, 2018; Cirlan, 2017; Fan et al., 2019; Green et al., 2007; Liu et al., 2016). Teachers' assessment practices can largely influence students learning, student motivation, student engagement with course materials, and teachers' actual teaching (Aitken, 2012; Brookhart & Nitko, 2015; McMillan & Moore, 2020; White, 2009).

As part of their professional responsibility, teachers need to use high-quality information in their assessment practices to provide a complete picture of students’ achievement (Tierney, 2014) and to make appropriate decisions about students learning outcomes (Brookhart, 2004; Brookhart & Nitko, 2018). For this, as scholars admit, teachers’ assessment practices should be aligned with assessment ‘ethics’- rules of behaviors, norms, and standards guiding assessment practices (Brookhart & Nitko, 2015; Thorndike et al., 1991). For instance, it has been found that good strategies teachers may employ to communicate about grading (Green et al., 2007; Pope et al., 2009), or respecting students in providing assessment feedback (standard assessment administration) (Bempechat et al., 2013; Colnerud, 2006; Ehrhardt et al., 2016; Horan & Myers, 2009), can largely improve student learning (Aitken, 2012) and their motivation (McMillan & Moore, 2020). Such assessment practices are perceived as ethical (Bergman, 2013, 2018, 2020; Cirlan, 2017; Fan et al., 2019; Liu et al., 2016; McGlory, 2013). Whereas using a few surprise items about topics that were not on the study guide (assessment development) (Brown & Harris, 2016; Green et al., 2007; Pope et al., 2009) or breaching the confidentiality and privacy of the students by publicizing the exam results (assessment communication) (Aitken, 2012) can hinder students' actual performance; hence considered as unethical. These factors demonstrate that ethics is focal and of prime significance in educational assessment (Johnson et al., 2008).

It is documented that perceptions of ethical issues in assessment could make it possible to understand how teachers’ biases and malpractices (ethical issues) can play a role in misinterpreting student achievements (Brookhart & Nitko, 2018). It is also evident that teachers' perceptions of assessment might affect their behavior and subsequent quality of teaching in their future endeavors (Maxwell & Schwimmer, 2016). Despite this, the practical application of ethical teaching and assessment across teacher education programs needs to be more extensive (Sanger & Osguthorpe, 2013; Schwartz, 2008). Exploring student teachers' perceptions of assessment ethics across university-based teacher education programs is relevant (Maxwell & Schwimmer, 2016).

A few studies have been reported to examine student teachers’ perceptions of ethical issues in assessment *(*Bergman, 2013, 2018; Cirlan, 2017; Fan et al., 2019; Liu et al., 2016; McGlory, 2013). Combined, these studies have utilized a series of scenarios of ethical assessment (Bergman, 2013; Bergman, 2018; Bergman, 2020; Cirlan, 2017; Fan et al., 2019; Liu et al., 2016; McGlory, 2013). However, as researchers admit (Liu et al., 2016), using a limited number of scenarios might only address some of the issues of assessment practices. Coupled with this gap, professional disagreement on ethical issues across these studies is evident (Johnson et al., 2017), though educators have reached some relative agreement. This lack of consensus can be explained by the effect of cultural differences on students' perceptions of ethics in assessment (Liu et al., 2016). For example, previous studies of student teachers (e.g., Fan et al., 2019) have found high disagreement among Chinese and Americans about the ethicality of using a few surprise items in the test. Chinese teachers considered this item highly ethical (90%), while for Americans, it was reported as considerably low (33%).

The present study furthers our understanding by qualitatively analyzing TEFL student teachers' perspectives on ethical issues in assessment across a few university-based teacher education programs in Iran (we selected senior TEFL students since this group is familiar with the basic tenets of assessment).

This study may contribute to the literature on assessment ethics in two aspects. First, given the cultural and contextual diversity of perceptions of ethical issues (Green et al., 2007; Johnson et al., 2017; Pope et al., 2009), additional research is needed to examine this area across various contexts and cultures (Fan et al., 2017; Liu et al., 2016; Johnson et al., 2017), particularly in the context of university-based teacher education programs. As such, the present study might shed more light on the cross-cultural issues of assessment ethics in teacher education. Second, in Iran, many student teachers enroll in mandatory undergraduate courses in university-based teacher education programs. Despite the significance of assessment ethics in this context, to my knowledge, no particular study has been conducted to examine student teachers' (specifically TEFL students) perceptions of ethical issues in assessment. The present study is a beginning attempt to attend to this void.

## Background of the study

### Theoretical background

Scholars have recognized the importance of ethical practices within classroom assessment (e.g., Pope, 2006; Pope et al., 2009; Popham, 2000). Classroom assessment is a process teacher, and students use to collect information from various sources about enhancing students learning and teachers' teaching for formative and summative purposes (Brookhart et al., 2020; McMillan, 2017). While “formative assessment is a way of assessing students' progress, providing feedback, and making decisions about further instruction, summative assessment is conducted after instruction, primarily as a way to document what students know, understand, and can do” (McMillan, 2017, p. 15). Despite the enormous influence of formative assessment on student learning, findings from some past studies on fairness and ethics in classroom assessment indicate that most of the assessment practices in higher education are summative-oriented (Ahmadi, 2021; Darabi Bazvand & Rasooli, 2022; Panadero et al., 2016; Rasooli et al., 2019). The assessment environment is heavily weighted in favor of final examinations (Ahmadi, 2021).

A couple of researchers recommended that ethical classroom assessment practices be consistent with two basic principles: “do no harm” (Taylor & Nolen, 2005) and “avoid score pollution” (Green & Johnson, 2010; Green et al., 2007). “Do no harm” requires that teachers make every effort to avoid causing harm to individuals in schools in general and students in particular (Green et al., 2007; Taylor & Nolen, 2005). “Avoid score pollution” refers to withdrawing from “any activity that enhances students’ scores on assessment without improving their actual mastery of the content (Green et al., 2007).

Based on these principles, some guidelines were developed (Green et al., 2007): *standardized test administration* (controlling the physical environment and the standard administrators’ behaviors), *multiple assessment opportunities* (multiple-choice, essay type, oral presentation, and the like), *communication about grading* (teachers communicate with the students about the test content, test method, test administration, test results, and the interpretation based on the test results) *grading practices* (the howness of scoring, returning practices of the final results or scores to the students (JCSEE (Joint Committee on Standards for Educational Evaluation), 2015, and *confidentiality* (keeping the results of test confidential). When teachers live up to these guidelines, their assessment practices are said to be relatively ethical, whereas violation of any of these guidelines renders them unethical (Green et al., 2007). These guidelines are considered good criteria for ethics-related studies in assessment. Specifically, these guidelines are the benchmark for the present study to frame doctoral students’ perceptions of their instructors’ ethical/ unethical practices within the university-based teacher education programs in Iran.

### Empirical studies on students’ perceptions of assessment ethics

Unlike sizable published research on students’ perceptions of assessment in higher education, until recently, very little has been reported on student teachers’ perceptions of assessment ethics across university-based teacher education programs (Bergman, 2013, 2018; Cirlan, 2017; Fan et al., 2019; Green et al., 2007; Liu et al., 2016). Furthermore, studies carried out in this area are relatively new in the literature (Gao et al., 2021). These studies have examined ethical issues in assessment across specific stakeholder groups, including university professors (Fan et al., 2017, 2019, 2020), in-service teachers (Pope et al., 2009), educational leaders (Johnson et al., 2008), and preservice teachers (Bergman, 2013, 2018; Cirlan, 2017; Fan et al., 2019; Green et al., 2007; Liu et al., 2016). Across these studies, the researchers have followed some ethical guidelines for student assessment, including bias/fairness, communication about grading, grading practice, confidentiality, standardized administration conditions, and multiple-assessment opportunities. Green and colleagues initially developed these guidelines.

The initial research on preservice teachers’ perceptions of ethical issues in assessment was carried out by Green and colleagues (2007). They analyzed 169 preservice and in-service teachers’ ethical perceptions and justification of a range of classroom assessment practices in the US by responding to some scenarios. Findings indicated strong agreement among the preservice teachers on fewer than half of the scenarios utilized in this study. A few years later, Bergman (2013) replicated the previous study with 264 undergraduate preservice teachers enrolled in a mandatory teacher education program at a large urban Midwestern university. Preservice teachers highly agreed on more than half of the scenarios (53%), which lends support to the findings from the previous research. Three years later, in a comparative study, Liu and colleagues (Liu et al., 2016) examined the same scenarios with 173 preservice teachers in the U.S. and 174 preservice teachers in China. Findings revealed that in different scenarios, the perceptions of Chinese and American respondents were divided. Additionally, it was indicated that the preservice teachers differed in their opinions on 22 out of 36 scenarios, whereas they had similar perceptions on 14 scenarios.

Collectively, these studies have used a couple of scenario-based items to judge the ethicality or unethicality of several assessment practices across different contexts and cultures. Researchers admit that using a limited number of scenarios might hardly address the ethical issues in assessment practices (Liu et al., 2016). The present study attempts to extend the previous studies by qualitatively examining student teachers’ perceptions of ethical issues in assessment across a few university-based teacher education programs in Iran. Thus, the following research questions were raised:

- RQ1: To what extent do Iranian TEFL teacher candidates perceive their teacher educators’ assessment practices as ethical/unethical?
- RQ2: To what extent are Iranian TEFL teacher candidates’ perceptions and experiences of assessment summative/formative-oriented?

### Assessment context in Iran

In Iran, university-based teacher education is implemented in some higher education universities and institutes under the auspices of the Ministry of Education (MOE) and the Ministry of Science, Research, and Technology (MSRT). Under a shared jurisdiction, in the 2010–2011 academic year, these two ministries established a new institution known as Teacher (Farhangian) University, practising the same policies as other nationwide universities. As a shared responsibility, MOE and MSRT control teacher candidates’ admission, preparation, and certification; universities must implement the policy mandates from the two ministries (Dadvand, 2015). As a part of their training program, student teachers should complete a constellation of undergraduate compulsory courses such as ‘teaching skills,’ ‘practicum,’ and ‘classroom management’ (Darki, 2006). Student teachers must manage the successful completion of field-specific courses towards specialization (Dadvand, 2015). After graduation, they are required to teach in the Office of Education for about two times the period of their education.

Concerning the assessment procedures in the Iranian teacher education programs, the grading system ranges from 0 to 20, and the passing criterion for undergraduate (BA) levels is 10, indicating that the evaluation is more objective-oriented. Assessment, in this context, is more theoretically-oriented (Tavana, 1994), depending more on examination and testing culture than any other assessment method, such as peer assessment, self-assessment, portfolio, and conferences (Brown et al., 2014). It is quite commonplace in university assessment that the educational system does not encourage peer work, and due to their inadequate knowledge of assessment, students themselves think that they cannot be fair in assessing their classmates (Ahmadi, 2021). Thus, teacher educators may feel uncomfortable with student-initiated assessment. In sum, Iran’s assessment culture is more in line with the authoritarian approach (Ahmadi, 2021) and instructors’ dominance (Bergmark & Westman, 2018).

## Method

### Research design

An inductive approach to thematic analysis (Clarke & Braun, 2017) was adopted to obtain thick and rich data from participants. Thematic analysis is a model in which patterns of meaning (themes) are identified, analyzed, and interpreted within qualitative data (Braun & Clarke, 2013; Clarke & Braun, 2017). This approach can “identify patterns within and across data concerning participants’ lived experience, views and perspective” (Clarke & Braun, 2017, p. 297). Since the researcher aimed to explore student teachers’ perceptions of assessment ethics, he employed inductive thematic analysis as the best method to suit this purpose.

### Participants

The study’s participants included 15 undergraduate student teachers enrolled in three university-based teacher education programs. The universities were located in three provinces in the central and southern parts of Iran. These sites were selected based on their geographical distribution. All the participants were male students aged 21 to 24 years, and their participation was voluntary across the three universities. They were senior student teachers majoring in TEFL and were selected based on criterion sampling. The criterion sampling method allows the researcher to identify and select information-rich cases (Miles & Huberman, 1994). Senior students were selected since the researcher assumed they might have had more to contribute to the richness of the data as they might have more theoretical knowledge of assessment concepts and principles.

### Ethical issues addressed in the study

Since in the present study, the researcher recruited human subjects (teacher candidates), he made concerted efforts to follow the ethical guidelines in conducting research. First, he explained the study’s objectives to the department heads and, getting permission from them, made initial contact with the teacher candidates at various times. Then he briefed them about the purpose of the study and provided them with informed consent (Punch, 1994). Having obtained the consent of those who agreed to participate in the study willingly, the researcher arranged a time with them to run the phone interview (see the next part for more information on this method). Before initiating phone interviews, efforts were made to ensure ease of communication with student teachers. At the onset of each interview, the interviewer’s style was courteous, friendly, and unbiased. To allow participants to feel at ease to express their perceptions and experiences openly, the researcher introduced the topic, relayed to them that the responses would be confidential*,* explained how the information would be used and informed them of the estimated length of the interview. Finally, he used a pseudonym for each participant to heighten the data’s anonymity.

### Instrument and data collection

The instrument used to collect the data was a semi-structured phone interview. This method allows the researchers to access geographically dispersed interviewees, greater flexibility for scheduling, and reduced costs (Lechuga, 2012). Phone interviews also have methodological strengths such as reduced distraction, perceived anonymity, and increased privacy for respondents (Lechuga, 2012). Due to Covid-19 limitations, the researcher could not adopt other sources and instruments (e.g., focused group, observation, and the like).

Having established a congenial context, the researcher initiated the interview to tap into the participant’s perceptions about the ethical issues in assessment. Further to audio-recording, the researcher hand-recorded the data to make sure the data would not be lost in case of inconvenience. The interview took place in the course of early January (2021). Each guided interview lasted 15 minutes and solicited student teachers’ perceptions of assessment ethics. An interview guide was used to make sure that the same data were gathered from the participants. The interview guide consisted of a prompt to structure the interview, but when needed, the interviewer also explored, probed, and asked additional questions to clarify and expand on a particular topic: *You are kindly invited to discuss your perceptions and experiences of real-life language assessment. You may verbalize your positive, negative, or neutral experiences with the language assessment.* The interview guide helped make interviewing more systematic and comprehensive by defining the issues to be explored (Patton, 1990). Depending on the participants’ initial responses, the researcher asked them to add any further points they desired or had failed to share during the interview. It is worth noting that the interviews were run in the students’ native language (Farsi) so that the participants could easily express their perceptions. Later, the researchers recruited two well-experienced translators to translate the transcripts into English. Finally, the researcher transcribed all the audio-recorded interviews verbatim to be analyzed later. The transcriptions were printed and double-checked.

### Data analysis procedures

A thematic content analysis was adopted in this study to analyze the data and extract themes relevant to the participants’ perceptions (Braun & Clarke, 2013; Patton, 2002). For this analysis, the researcher followed a six-step process recommended by Braun and Clarke (2013). In the first step, the researcher read and reread the transcripts so much that he became acquainted with them. In the succeeding stage, the researcher tried to extract some properties and generate the initial codes. Next, high-degree co-occurrences of some codes that represented similar concepts were combined into broader themes. For example, ‘content under-representation ‘multiple assessment opportunities and ‘surprise items’ were coded together and were, thus, subsumed under the broader theme of ‘ethical issues in assessment development. Afterwards, candidate themes were reviewed against the entire data set and the coded data. The themes were defined, which were pertinent to ‘ethical issues in assessment development,’ ‘ethical issues in exam administration,’ and ‘ethical issues in assessment scoring and communication. ‘Finally, the researcher wrote up the analysis and ensured the credibility of the findings by employing member checking. For this purpose, the contents and interpretations were reviewed meticulously by sending a copy of the final categories and excerpts to the participants through email to check if they represented their intended meanings. In general, they affirmed the interpretations and the findings.

## Findings

**Fig. 1:**
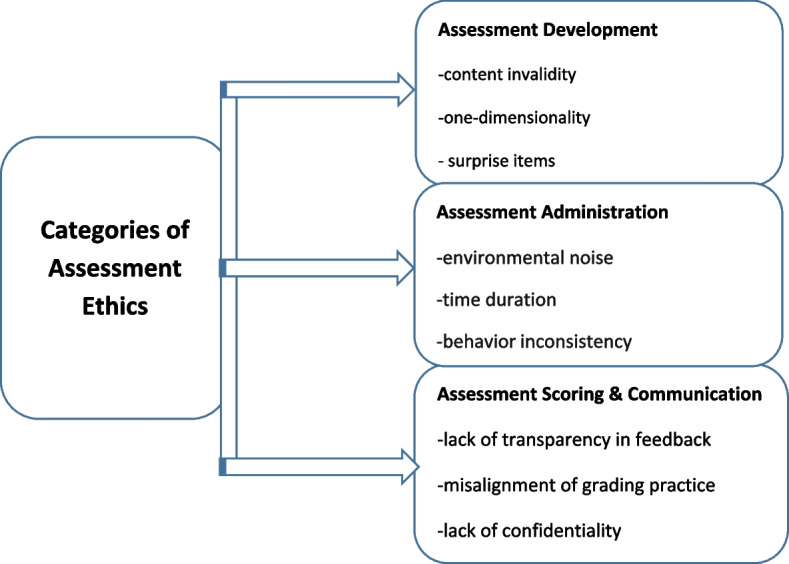
Categories of assessment ethics

The analysis of student teachers’ phone interviews revealed three overarching themes of assessment ethics, along with their corresponding sub-categories related to (a) assessment development (i.e., content invalidity, one-dimensional assessment, and surprise items), (b) assessment administration(i.e., timing, noise, and inconsistency in educators’ behaviors), and (c) assessment scoring and communication (i.e., lack of transparency in feedback provision’ ‘misalignment of grading practice,’ and ‘ breaching confidentiality in grade communication’). The major guidelines and their corresponding components are presented in what follows. Figure 1 displays the schematic representation of the categories for each theme pertinent to assessment ethics.

### Ethical issues in assessment development

Most of the participants in this study complained about the teacher-made final exam questions in terms of content validity. They argued that some of the test items developed by the teacher educators were quite easy, and some were unduly difficult and may not tap into the real abilities of teacher candidates. To confirm this, one of the participants complained:

*Test difficulty needs to be sufficiently taken into account in classroom assessment. In reality, sometimes we find some easy questions, sometimes reasonable, and most of the time unduly difficult. As a bitter experience, last semester, one of the final exams was so difficult that most of my classmates could not pass. More surprisingly, we could not express our dissatisfaction with the test items(S7).*

Further to the test difficulty, participants claimed that most of the final exam questions that teacher educators develop might be severely prone to the problem of content and construct underrepresentation. They commented that sometimes test tasks were partially commensurate with the objectives and requirements of syllabuses of TEFL in teacher education. By way of illustration, one of the participants mentioned:

*Unluckily, most teacher educators need to pay more attention to content validity. Sometimes, they extract some questions from course books, pile them up, and give them to students. For example, in one case, when I checked out the questions one by one, I found that just around 60 percent of the questions were embedded in the course materials(S3).*

Furthermore, under the previous statements, some university students were concerned about face validity in that this principle usually gains scant attention from teacher educators. To corroborate this, one of the participants complained:

*Sometimes the tests are designed and presented in an unacceptable form. For example, I have experience with tests whose items needed to be typed in proper font size; there were many spelling and grammar mistakes in the test items, and there needed to be more space to write answers comfortably(S9).*

Participants also perceived teacher educators’ failure in employing **‘**multiple assessment procedures’ as unethical. They commented that assessing students’ ability through a multiple-choice test or essay-type examination may not tap into the students’ real abilities. This is because some students may perform better on multiple-choice questions, while others feel at ease with essay-type ones. To counterbalance this, instructors would use multiple assessment procedures. These factors may highly affect students’ performance on language assessment. One of the participants railed about this issue:

*Developing multiple assessment methods can be found promising and motivating for test-takers since different students with different individual differences may perform differently on different tests. For example, some students act well on multiple-choice tests though other test takers may perform better on open-ended tests due to differences in their cognitive styles(S3).*

Another important ethical issue of concern for teacher candidates is using ‘surprise items.’ The importance of this aspect of language assessment was recognized by most of the teacher candidates. They lamented that most of the time, they were not satisfied with their scores, not because of their inability in test performance but due to unfamiliar material and evaluation methods. For example, one of the participants in this regard emphasized that:

*Concerning this issue, I believe that refusing to take a test is nearly impossible. But, we, students, have the right to avoid taking a test when we see that some items are unfamiliar to us(S12).*

In addition, the data revealed that sometimes there needs to be more transparency in the syllabi and educational materials, which may lead to surprise items. One of the interviewees backed up this claim by asserting:

*What I have experienced in some courses is the need for more clarity in the materials. Some teacher educators need to clarify the contents for the students. As a consequence, they may include some surprise items in the exams. What makes the story more upsetting is that you cannot refuse to take the test. Refusing to take the test equates to dropping the course(S1).*

### Ethical issues in assessment administration

The second major category perceived by the participants as unethical is related to issues in assessment administration. The most important factors contributing to this guideline are ‘environmental noise,’ ‘time duration,’ and ‘inconsistency in educators’ behavior. ‘Concerning noise, the predominant view among the informants was that they had been disadvantaged by some noises made by invigilators’ behaviors (e.g., noises to page the time for students with different fields of study sitting in the same exam hall and invigilators’ noises while talking to each other).

As participants proclaimed, although special measures were taken to minimize the chances for cheating, like designing samples A, B, and C or allowing every student with a special major to sit next to those with a different major, this introduced another sort of inconvenience into exam condition. Since each field of study has its time budgeting, noises from paging the time limit for different student groups (fields) distracted students’ attention and introduced systematic bias into testing conditions. For example, one of the participants lamented about noises made by paging the time:

*Students of different fields of specialization can be placed in the same place where the exam is administered. However, each field of study has its time budget, and when exam administrators page each group’s time, it can distract other students’ attention*. *This occasionally happens while sitting for the final exams(S4).*

Furthermore, student teachers expressed disdain about noises made by educators when sitting for a final exam. They believed that this behavior might compromise the fairness of the assessment. One of the participants, for example, referred to an unwelcome experience while taking the final exam.

*I have a bitter experience with assessment administration issues. I remember once, in one particular exam hall, one of the invigilators made many noises, talking over the phone and distracting our attention. When I later raised the issue with my classmates sitting in other halls, I found that this problem did not happen to them. This may breach the fairness of the test(S6).*

Further to the noise, some students pointed out that inconsistencies in time allocation were frustrating when they experienced that the test developer was inconsiderate in comparing the content of the exam questions they developed against the time allocated to finish them; sometimes, the allocated time was hardly sufficient for them to finish. One of the participants complained about the time above factor:

*One of the teachers allocated only 60 minutes for a lengthy exam on general linguistics. When we complained about the time, he commented that he did not want to provide an opportunity for some students to cheat. This comment upset us, and we perceived it as unethical(S11).*

Another important issue raised by a couple of teacher candidates about assessment administration is ‘inconsistency in educators’ behaviors’ regarding unclear instructions. They complained that sometimes instructors or invigilators breached the equality principle by responding to a group of students’ questions about unclear instructions while disregarding other counterparts. To corroborate this, one student expressed disdain:

*Once my classmates and I were sitting for a final exam on research methods, and one of the questions needed to make more sense to most of us. It hindered students’ understanding. Upon a student’s request, the teacher invigilator explained the question to him, and when we asked him to do so, he announced that the questions were clear and that invigilators were not allowed to explain the questions. We were disappointed(S13)*.

### Ethical issues in scoring practice and communication

The last important principle receiving considerable attention and perceived by the participants as unethical is ‘scoring practice and communication’. This major category included ‘lack of transparency in feedback provision’ (communication about grading), ‘misalignment of grading practice,’ and ‘lack of confidentiality in grade communication. ‘In summative assessment, transparency in feedback refers to teachers’ communication with the students about the exam results objectively and with ease. The predominant view among participants was that, for the most part, teacher educators did not provide any feedback about the exam results, and if they did, their feedback was not transparent. Therefore, as they claimed, across a summative assessment, it was their responsibility to return the marked papers, attend to any miscalculation of the scores, explain clearly to the students the exam criterion, and respond to potential misunderstandings. As an example, one of the university students opined:

*In most of the end-of-the-semester exams at my university, teacher educators still need to return the corrected papers. It is so natural and normal that students have accepted this as true. I believe the exam questions should be returned to the test takers. In that case, the test takers might understand their performance comprehensively, and sometimes they can learn from the error correction feedback given by teachers. Sometimes, teachers miscalculate the total scores(S10).*

In addition to feedback provision, providing a detailed report card is critical in enhancing the ethics of assessment, especially within classroom assessment (Tierney, 2013). Most participants lamented the lack of presenting a detailed and full report card concerning score descriptors: They claimed that a multi-dimensional report card represents the students’ performance on different sections of a test or final examination. One of the participants stated:

*The report card should be based on the multidimensionality here; representative report cards based on the performance on different test sections should be provided for the candidates. Their reporting is one-dimensional, giving a total score. It is a good idea to assign the test score to each component of the test(S14).*

Student teachers also complained about teachers’ inconsideration of providing transparent exam criteria and content. This factor can confuse and demotivate students to prepare well for the final examinations. One of the students complained about this issue:

*Sometimes, instructors extract some questions from course books, pile them up, and administer them to students. The content of these items may not represent the course syllabus. The reason for this is that we need to know exactly based on which criterion the questions have been developed(S15).*

The participants also perceived the misalignment of grading practices in dealing with students’ efforts as unethical. Given that scores are supposed to be the true reflection of students’ actual performance in any exam lowering or raising students’ grades due to their efforts or class attendance may pollute their actual performance (Green et al., 2007; Tierney, 2014). While it is the case, participants in this study complained that university instructors counted students’ non-performance factors, such as their efforts and disruptive behavior, as part of students’ final assessment. To confirm this, a student teacher lamented:

*Once I remember that in an end-of-semester exam, one of my teachers reduced my course grade from 18 to 16 because he believed that although my paper showed I had mastered the course objectives, you have not participated in-class activities during the semester(S2).*

‘Confidentiality’ was another essential requirement of grade communication in assessment perceived by the participants as unethical. In ‘confidentiality’, teachers needed to pay particular attention to students’ privacy and keep the results of tests confidential (Green et al., 2007). That is, test results should not be announced and revealed to anyone who does not have the legitimacy to be informed about test scores and results (JCSEE (Joint Committee on Standards for Educational Evaluation), 2015). Concerning this issue, a teacher candidate commented:

*Publicizing the students’ scores in front of the class is unethical. Teacher educators should not give out students’ scores in class or announce them publicly. They can give them directly to students themselves(S9).*

## Discussion

Exploring student teachers’ perceptions of assessment ethics in teacher education is construed as focal (Maxwell & Schwimmer, 2016). While enormous research has been conducted on assessment in higher education (e.g., Horan & Myers, 2009; Panadero et al., 2016), until recently, little has been written on student teachers’ perceptions of assessment ethics across university-based teacher education programs (Bergman, 2013, 2018; Cirlan, 2017; Fan et al., 2019; Green et al., 2007; Liu et al., 2016). Additional research is needed to conceptualize assessment ethics in teacher education. To add to the assessment literature, the present study drew on qualitative data to shed some light on how student teachers perceive issues pertinent to assessment ethics in the context of teacher education. This study’s findings revealed two interrelated factors related to assessment ethics. First, it was indicated that teacher candidates’ perceptions of assessment ethics were mainly pertinent to unethical practices. Secondly, it was revealed that student teachers’ perceptions of assessment were relatively summative-oriented. Findings for these two aspects of assessment ethics are discussed in line with the research questions.

The first research question was formulated to investigate the ethicality of the assessment practices of teacher educators as perceived by student teachers of TEFL. Findings revealed three aspects of unethical assessment practiced by teacher educators, including (a) assessment development, (b) assessment administration, and (c) assessment scoring and communication. Regarding ethical issues related to assessment development, participants commented that these issues were mainly pertinent to test validity. Not quite surprisingly, participants’ comments revealed that sometimes examination questions teacher educators develop for the final evaluation of the student teachers are partially commensurate with the objectives and requirements of the subject matter syllabuses of TEFL. Although validity is integral to and has huge effects on the accuracy of test results (Davies & Elder, 2005), within the Iranian context, teacher educators rarely exercise validation. Practicing validation is conceived as highly complex, demanding, and time-consuming by university teachers (Davies & Elder, 2005; Messick, 1994, 1996). Another important issue perceived by student teachers as unethical in assessment development is ‘multiple assessment opportunities. This guideline is significant and has been substantially perceived as unethical in past research (e,g. Green et al., 2007; Liu et al., 2016; Tierney, 2013). One reason for this study’s finding may go to a lack of professional development training courses in educational assessment (Plake & Impara, 1997; Stiggins, 1999). Thus, it is recommended that teacher educators instill in their assessment development multiple types of assessment to address the students’ individual differences.

The second major unethical guideline highlighted by most of the participants is assessment administration’. In this regard, most of the student teachers claimed that while sitting for the final exam, they were incessantly interrupted by unending noises made by invigilators, although some students may be less vulnerable to environmental noises than others. Participants in the present study complained that teacher educators needed to attend to this important area of assessment ethics. It can be justified by teacher educators’ inadequate knowledge of ethical and fairness principles (Darabi Bazvand & Rasooli, 2022) or possibly due to students’ lack of power to criticize this assessment malpractice (Ahmadi, 2021).

Finally, concerning the unethicality of ‘assessment scoring and communication’, the present study’s findings, consonant with prior literature (Darabi Bazvand & Rasooli, 2022; Rasooli et al., 2019; Tierney, 2015), identified ‘misinterpretation of grading’ as a recurrent theme of unethical/unfair assessment. For example, participants disdained that their instructors needed clear information about the assessment criteria and mentioned that the exam content needed to be adjusted to the course syllabus. The reason for providing transparent feedback is that instructors under social and psychological pressure may be mistaken in correcting students’ papers by miscalculating or misunderstanding students’ final grades. Thus, instructors’ feedback may address such misunderstandings and result in assessment fairness (Popham, 2017). Therefore, in line with scholars (Alkharusi et al., 2011; Lizzio & Wilson, 2008; McMillan, 2011; Popham, 2017), it is suggested that university teachers’ feedback for grading be treated with care and follow assessment standards.

The second research question was raised to examine the extent to which Iranian TEFL teacher candidates' assessment experiences were summative or formative- oriented. The study’s findings indicated that assessment of student teachers is more in concert with teachers’ dominance (Bergmark & Westman, 2018) and the authoritarian approach to student assessment (Ahmadi, 2021). That is, assessment is, for a great part, teacher-initiated and summative- oriented. This is not surprising since the assessment milieu in Iran is heavily dominated by final examinations and is more objectively-oriented (Brown et al., 2014). This finding might be consistent, albeit partially, with previous national (e.g., Ahmadi, 2021; Darabi Bazvand & Rasooli, 2022; Rasooli et al., 2019; Yan and Cheng, 2015) and international scholarship (Panadero et al., 2019). In the context of Iran, for example, Ahmadi (Ahmadi, 2021) concluded that students would hardly claim responsibility for voluntary homework and projects, so they may avoid completing them.

Furthermore, regarding university assessment, it is quite commonplace that the educational system in Iran does not encourage peer work, and due to their inadequate knowledge of assessment, students themselves think that they cannot be fair in assessing their classmates (Ahmadi, 2021). Given the usefulness of adopting formative assessments to enhance EFL students’ “academic motivation, attitude toward learning, test anxiety, and self-regulation” (Ismail et al., 2022) in Iran, teacher educators are recommended to instil formative assessment in their pedagogical practices (p.19). Internationally, this finding may reflect that of Yan (Yan and Cheng, 2015), who proclaims that students’ ideologies and identities have been shaped by examination culture in a way that may lead to students’ resistance to change.

## Conclusion

The present study explores student teachers’ perceptions of ethical issues in university-based teacher education programs. The study’s findings revealed that assessment is never neutral (Biesta, 2009) and needs to be understood in light of such ethical factors and issues as assessment development, assessment administration, and assessment scoring and communication. Given the results, progressive developments in teacher education are encouraged. Thus, professional development programs may instil in their assessment courses discussion on assessment ethics to increase teachers’ awareness of this phenomenon in teacher education (Gao et al., 2022).

Findings from the present study can shed some light on the professional development of teachers and may add to the conceptualization of ethics in assessment both in Iranian universities and in relatable contexts elsewhere. However, the transferability of the findings should be taken with care due to the particularity of the teaching, learning, and assessment culture in Iran. The higher education system in this country is top-down, where authorities in charge make decisions, and universities are required to implement the government’s pedagogical policies (Brown et al., 2014). Teachers in Iran (teacher educators included), thus, might have little or no control over the content of their instruction since the curriculum, hours of instruction, and method(s) of assessment are defined from the top for teachers, students, and schools (Dadvand, 2015). Therefore, any interpretation of Iranian student teachers’ perceptions about teacher educators’ assessment quality should be made with care.

Further to the assessment culture influencing the generalization of the findings, due to logistic issues and Covid-19 limitations, the present study adopted phone interviews as the data collection method. Across open-ended questionnaires and phone interviews, a lack of direct communication with participants may result in subjectivity and inaccuracy in the coding analysis (Gao et al., 2022). Thus, it is suggested that future researchers (in post-Covid 19) employ face-to-face interviews to access thick descriptions of the data and, as such, make a better interpretation of the findings. Finally, the present study recruited teacher candidates of TEFL to explore their perceptions of assessment ethics. Since disciplines might have different values or assessment standards, future researchers can replicate this study with student teachers from other departments and fields of study. Additionally, researchers can accrue more comprehensive data about assessment ethics from other stakeholder groups, such as in-service teachers, university professors, and university students.

### Notes

Throughout the manuscript, I have used the expressions “teacher candidate and student teacher interchangeably.”

## Data Availability

Following the principle of confidentiality, the researcher prefers to keep the data.

## Abbreviations

BA: Bachelor of Art
MOE: Ministry of Education
MSRT: Ministry of Science, Research, and Technology
TEFL: Teaching English as a Foreign Language
